# Patterns of electronic cigarette, conventional cigarette, and hookah use and related passive exposure among adolescents in Kuwait: A cross-sectional study

**DOI:** 10.18332/tid/123499

**Published:** 2020-07-07

**Authors:** Ali Esmaeil, Ahmed Alshammasi, Waad Almutairi, Abdullah Alnajem, Dalal Alroumi, Mohamad Ali, Abdullah Redha, Maram Alhussaini, Ali H. Ziyab

**Affiliations:** 1 Faculty of Medicine, Kuwait University, Kuwait City, Kuwait; 2 Department of Community Medicine and Behavioral Sciences, Faculty of Medicine, Kuwait University, Kuwait City, Kuwait

**Keywords:** electronic cigarettes, hookah, cigarettes, secondhand smoke, adolescents

## Abstract

**INTRODUCTION:**

Use of tobacco products among adolescents is a major global public health concern. Given the changing landscape of tobacco product use and the lack of epidemiologic data to inform tobacco prevention and control strategies in Kuwait, this study sought to estimate the prevalence and patterns of electronic cigarette (e-cigarette), conventional cigarette, and hookah use among adolescents in Kuwait. Moreover, exposure to secondhand smoke (SHS) and secondhand aerosol (SHA) from e-cigarettes was assessed.

**METHODS:**

This cross-sectional study enrolled high school students (n=1565; 16–19 years) across Kuwait. Current (past 30-day) use of e-cigarettes, conventional cigarettes, and hookah were assessed through self-reported data. Additionally, current (past 7-day) exposure to SHS and SHA in households and public places were ascertained. Associations were evaluated using Poisson regression, and adjusted prevalence ratios (APRs) and 95% confidence intervals (CIs) were estimated.

**RESULTS:**

Overall, 26.4% (402/1525), 25.1% (383/1525), and 20.9% (318/1525) of the study participants were current e-cigarette users, conventional cigarette smokers, and hookah smokers, respectively. Current use of any tobacco product was reported by 35.1% (535/1525) of the total study participants. The prevalence of concurrent triple use of ‘e-cigarettes, conventional cigarettes, and hookah’ was estimated to be 12.8% (195/1525). Also, among the study participants, 41.9% (619/1479) were exposed to household SHS, 32.0% (469/1465) were exposed to household SHA, and 62.2% (916/1472) were exposed to SHS and/or SHA in public places. Male adolescents were more likely than females to be current e-cigarette users (APR=5.19; 95% CI: 4.09–6.57), conventional cigarette smokers (APR=5.42; 95% CI: 4.26–6.90), and hookah smokers (APR=3.43; 95% CI: 2.72– 4.32).

**CONCLUSIONS:**

A substantial proportion of adolescents in Kuwait are currently using tobacco products and being exposed to SHS/SHA. The findings emphasize the need to continue monitoring all forms of tobacco product use among adolescents and to strengthen tobacco prevention and control programs.

## INTRODUCTION

Tobacco smoking remains a major global public health concern that has been ranked as the second leading preventable cause of premature death and disability^1^. In 2017, smoking accounted for about 7.1 million deaths and 182 million disability-adjusted life-years (DALYs) worldwide^1^. Although global trends indicate that tobacco smoking prevalence has declined during the past 30 to 50 years, the concurrent population growth and aging contributed to an increase in the absolute number of smokers, leading to higher smoking-attributable mortality^2,3^. Despite the global declining trends, wide disparities in smoking across geographical regions and demographic subgroups still exist^4^. Hence, public health efforts are needed to address such disparities and to control smoking.

Adolescents have been the target and critical market of the tobacco industry given that nearly 90% of current smokers have started smoking before their 18th birthday^5^. Moreover, given the strong addictive nature of nicotine, smoking during adolescence tends to persist into adulthood^5^. Although public health efforts have been successful in reducing and controlling cigarette smoking initiation and use among adolescents^2,4^, the rapid emergence of alternative smoking modalities among youth is a current major global public health challenge. Hookah (also known as waterpipe or shisha) smoking and electronic cigarette (e-cigarette) use are ever increasing in popularity, especially among adolescents due to the wide variety of used flavors and the misperception of being less harmful than conventional cigarettes^6-9^. For instance, among high school students in the United States, e-cigarette use increased from 11.7% to 20.8% from 2017 to 2018^8^. In addition to their adverse health effects, hookah and e-cigarette use during adolescence have been shown to be associated with increased subsequent use of conventional cigarettes and illicit drugs^10-13^; hence, potentially, undermining the achieved success in reducing smoking in youth.

No less than active smoking, exposure to secondhand smoke (SHS) is associated with detrimental health effects for children and nonsmoker adults. SHS exposure among infants and children is related to sudden infant death syndrome, respiratory infections, ear infections, and asthma attacks. In addition, it may be a risk factor for coronary heart disease, stroke, and lung cancer in adult nonsmokers^14,15^. Global estimates from 2004 showed that 40% of children, 35% of female non-smokers, and 33% of male non-smokers were exposed to SHS^16^. On the other hand, with the recent surge in e-cigarette use, exposure to secondhand aerosols (SHA) from e-cigarettes could be substantial. In 2015, 29% of high school students in the United States reported SHA exposure^17^. E-cigarette aerosols contain ultrafine particulate matter, volatile organic compounds, and heavy metals, which make exposure to SHA from e-cigarettes potentially harmful^18^. Exposure to SHA has been linked with increased susceptibility to initiate tobacco smoking and e-cigarette use among youth who never smoked^19^.

Although Kuwait ratified the World Health Organization Framework Convention on Tobacco Control (WHO FCTC) in 2006 and issued and implemented several laws to control tobacco use (e.g. banning smoking in public places, workplaces, and on public transportation; prohibiting marketing and promotion of tobacco products; requiring warning labels on tobacco product packages), the burden of tobacco smoking remains substantial in Kuwait. For instance, among adults in Kuwait, the prevalence of daily smoking of tobacco has been estimated to be 23.2% (20.9–25.6%) among men and 5.2% (4.1– 6.5%) among women^4^. Also, of 195 countries and territories, Kuwait was one of only four countries that had significant annual increases in tobacco smoking prevalence between 2005 and 2015 among adults^4^. Among adolescents, the Global Youth Tobacco Survey (GYTS) conducted in 2016 among school adolescents aged 13–15 years estimated that 15.4% of adolescents in Kuwait were current cigarette smokers, with more boys than girls reporting current cigarette smoking (23.2% vs 8.3%)^20^. In addition, it has been estimated that nearly 50% of middle and high school students are exposed to household SHS in Kuwait^21^. Given the changing landscape of tobacco product use and the rapid emergence of e-cigarette and hookah use among adolescents, and due to the clear lack of empirical knowledge in Kuwait, there is a need for epidemiologic data to guide future policies and public health strategies that aim to protect this vulnerable population. To this end, this study sought to estimate the prevalence and patterns of e-cigarette, conventional cigarette, and hookah use among adolescents in Kuwait, and to assess the extent of exposure to SHS and SHA in households and public places among adolescents.

## METHODS

### Study setting, design, and participants

Geographically, Kuwait is divided into six governorates, and the school districts follow a similar geographical distribution. Education in Kuwait is mainly provided by free public schools funded by the state and, to a lesser extent, by private schools. The education system can be divided into four stages, namely, kindergarten, elementary school (1st–5th grade), middle school (6th–9th grade), and high school (10th–12th grade), and in the latter three stages, the students are segregated by sex. Schooling is compulsory for all children aged 6 to 14 years.

This cross-sectional study enrolled schoolchildren (n=1565) attending public high schools (11th and 12th grade) throughout the State of Kuwait, which included children aged between 16 and 19 years. The schoolchildren were enrolled in the study during the second semester of the 2018–2019 school year (January to May 2019). A stratified twostage cluster sampling method was used to select a representative study sample of schoolchildren from a random sample of schools. At the time the study was initiated, there were 139 public high schools in Kuwait enrolling approximately 60663 students (26692 males and 33971 females). From a list obtained from the Ministry of Education, Kuwait, of all public high schools stratified by school district and sex, schools were randomly selected using randomly generated numbers. Proportional allocation was used to determine the number of participants needed from each school district by estimating sex-stratified weights relative to the student body size in each given school district. In total, 14 schools served as the recruitment venues for enrolling the required sample size. The study was approved by the Health Sciences Center Ethics Committee for Student Research at Kuwait University (No. 750). Written informed consent was taken from each student. As per the waiver obtained from The Ethics Committee, no consent was sought from the parents.

### Questionnaire and variable definitions

A study-specific questionnaire that adapted questions from the National Youth Tobacco Survey (NYTS) questionnaire^8^ was developed, and self-completed by the students. The questionnaire gathered information on sociodemographic data, e-cigarette use, conventional cigarette use, hookah use, and exposure to SHS and SHA. It is important to mention that the GYTS instrument^22^ is as valid as the NYTS instrument and has been used at wider global settings; however, using the NYTS allowed us to make comparisons with recent reports on rapidly emerging tobacco products^8,23^.

Statements explaining each type of tobacco product were provided in a logical manner following the NYTS questionnaire^8^. Students were asked to report if they have ever tried using e-cigarettes, smoking conventional cigarettes, and smoking hookah. Moreover, frequency of using the aforementioned tobacco products during the past 30 days, in terms of number of days, was reported. Current conventional cigarette smoking was defined as any smoking of cigarettes in the past 30 days^8^. Similarly, current hookah smoking and e-cigarette use were defined as any use in the past 30 days^8^. Moreover, tobacco product use for ≥6 days, ≥20 days, and every day in the past 30 days were defined. Among those who are current (any use in past 30 days) users of e-cigarettes, conventional cigarettes, and/or hookah, single, dual, and triple use status were determined.

Students were asked to answer three separate questions regarding SHS and SHA exposure that were adapted from the NYTS questionnaire^8^. Exposure to household SHS was ascertained by asking: ‘During the past 7 days, on how many days did someone smoke tobacco products in your home while you were there?’. Household SHA exposure was defined by asking: ‘During the past 7 days, on how many days did someone smoke e-cigarettes in your home while you were there?’. While public exposure to SHS/SHA was determined by asking: ‘During the past 7 days, on how many days were you exposed to the smoke of tobacco products or e-cigarettes in public places (e.g. school, restaurants, coffee shops etc.)?’. Current exposure to household SHS, household SHA, and public SHS/ SHA was defined as any exposure in the past 7 days.

### Statistical analysis

All statistical analyses were carried out using SAS 9.4 (SAS Institute, Cary, North Carolina, USA). The statistical significance level was set at α=0.05 for all association analyses. Descriptive analyses were carried out to determine the frequencies and proportions of categorical variables. To assess whether the analytical study sample (n=1525; sample of participants with complete information on status of current e-cigarette, conventional cigarette, and hookah use) was representative of the total enrolled study sample (n=1565), we compared proportions of categorical variables across these two samples using chi-squared (χ^2^) tests.

Prevalence estimates of using e-cigarettes, conventional cigarettes, and hookah were determined in the total sample and stratified by sex, according to different frequencies of use in the past 30 days. Moreover, prevalence of currently using ‘e-cigarettes only’, ‘cigarettes only’, ‘hookah only’, ‘e-cigarettes + cigarettes’, ‘e-cigarettes + hookah’, ‘cigarettes + hookah’, ‘e-cigarettes + cigarettes + hookah’ were estimated in the total sample and stratified by sex. Similarly, prevalence of ‘any tobacco product use’ (i.e. current use of e-cigarettes, cigarettes, and/or hookah) and ‘≥2 tobacco products’ (i.e. current use of two or more of the aforementioned tobacco products) were estimated. Furthermore, prevalence estimates of exposure to household SHS, household SHA, and public SHS/SHA were reported as being exposed for ≥1 day, ≥3 days, and every day in the past 7 days.

Adjusted associations were assessed by applying a modified Poisson regression with robust variance estimation using the GENMOD procedure in SAS 9.4 to estimate and infer the adjusted prevalence ratios (APRs) and their 95% confidence intervals (CIs)^24^. We evaluated the associations of sex, age, and maternal and paternal educational level (exposure variables) with current use of e-cigarettes, conventional cigarettes, hookah, and current exposure to household SHS and SHA, and exposure to SHS/SHA in public places.

## RESULTS

In total, 1575 high school students (732 boys and 843 girls) were invited to participate, and 1565 (729 boys and 836 girls) were enrolled in the study (response rate: 99.4%). The analytical study sample (n=1525; restricted to participants with complete information on status of current e-cigarette use, conventional cigarette smoking, and hookah use) and the total study sample (n=1565) were similar in all characteristics investigated (Table 1). The enrolled study participants were mainly aged between 16 and 18 years, with 54.6% of the participants aged 17 years. The majority (96.5%) of enrolled adolescents were of Kuwaiti nationality. Majority of the participants’ mothers (42.5%) and fathers (47.2%) reported to have a bachelor’s degree or higher (Table 1).

**Table 1:** Characteristics of the total study sample and the analytical study sample

*Characteristics*	*Total study sample (n=1565) n (%)*	*Analytical study sample (n=1525)^a^ n (%)*
**Sex**		
Female	836 (53.4)	831 (54.5)
Male	729 (46.6)	694 (45.5)
**Age** (years)		
≤16	386 (25.1)	381 (25.4)
17	843 (54.7)	819 (54.6)
≥18	311 (20.2)	300 (20.0)
Missing, n	25	25
**Maternal education**		
Middle school or less	146 (9.6)	142 (9.6)
High school	382 (25.05)	369 (24.8)
Diploma^b^	352 (23.1)	344 (23.1)
Bachelor’s degree or higher	645 (42.3)	631 (42.5)
Missing, n	40	39
**Paternal education**		
Middle school or less	131 (8.6)	126 (8.5)
High school	377 (24.8)	363 (24.5)
Diploma^b^	297 (19.6)	293 (19.8)
Bachelor’s degree or higher	714 (47.0)	698 (47.2)
Missing, n	46	46

a Refers to the sample of participants with complete information on status of current electronic-cigarette use, cigarette smoking, and hookah use (i.e. excluding 40 subjects with incomplete information regarding tobacco products use).

b Refers to a two-year associate degree post high school.

Prevalence estimates of e-cigarette, conventional cigarette, and hookah use are shown in Table 2. Overall, 26.4% (95% CI: 24.2–28.6%), 25.1% (22.9–27.3%), and 20.9% (18.8–22.9%) of the study participants were current (any use in the past 30 days) e-cigarette users, current conventional cigarette smokers, and current hookah smokers, respectively. The highest frequent use (i.e. ≥20 days) and daily use during the past 30 days were reported for conventional cigarettes (frequent: 15.5%; daily: 12.9%), followed by e-cigarettes (frequent: 8.1%; daily: 5.7%). Furthermore, use of e-cigarettes, conventional cigarettes, and hookah was more common among males compared to females (Table 2).

**Table 2:** Prevalence of e-cigarette, conventional cigarette, and hookah use according to frequency of use in the total analytical sample and stratified by sex

*Frequency*	*Total analytical sample (n=1525)*	*Males (n=694)*	*Females (n=831)*	p*
	*% (n)*	*% (n)*	*% (n)*		
**E-cigarette use**				
Ever use	37.4 (570)	62.7 (435)	16.3 (135)	<0.001
Any use in past 30 days	26.4 (402)	46.8 (325)	9.3 (77)	<0.001
≥6 days in past 30 days	14.3 (218)	27.4 (190)	3.4 (28)	<0.001
≥20 days in past 30 days	8.1 (124)	15.6 (108)	1.9 (16)	<0.001
Every day in past 30 days	5.7 (87)	11.5 (80)	0.8 (7)	<0.001
**Conventional cigarette use**				
Ever use	42.5 (648)	67.9 (471)	21.3 (177)	<0.001
Any use in past 30 days	25.1 (383)	45.1 (313)	8.4 (70)	<0.001
≥6 days in past 30 days	18.9 (288)	36.7 (255)	4.0 (33)	<0.001
≥20 days in past 30 days	15.5 (237)	31.4 (218)	2.3 (19)	<0.001
Every day in past 30 days	12.9 (196)	26.1 (181)	1.8 (15)	<0.001
**Hookah use**				
Ever use	37.1 (566)	57.6 (400)	20.0 (166)	<0.001
Any use in past 30 days	20.9 (318)	33.9 (235)	10.0 (83)	<0.001
≥6 days in past 30 days	9.4 (144)	15.7 (109)	4.2 (35)	<0.001
≥20 days in past 30 days	4.3 (65)	7.1 (49)	1.9 (16)	<0.001
Every day in past 30 days	3.7 (56)	6.5 (45)	1.3 (11)	<0.001

* p-values comparing male and female proportions using chi-squared tests.

Frequencies of single, dual, and triple current use of e-cigarettes, conventional cigarettes, and hookah were determined (Table 3). Among the total study sample, hookah smoking only (2.4%). The concurrent dual the prevalence estimates of current e-cigarette use current use of ‘e-cigarettes + cigarettes’ was the most only and current conventional cigarette smoking only common (6.0%) among the total study participants were similar (4.0% vs 4.2%) and higher than current followed by the dual current use of ‘e-cigarettes + hookah’ (3.5%). The prevalence of concurrent triple current use of ‘e-cigarettes, cigarettes, and hookah’ was estimated to be 12.8% (Table 3). Moreover, the current use of any tobacco product and ≥2 tobacco products was reported by 35.1% and 24.5% of the total study participants, respectively. In general, male participants reported higher use of tobacco products, across almost all types and combinations, compared to female participants. For instance, the prevalence of concurrently using ‘e-cigarettes, cigarettes, and hookah’ among males was estimated to be 23.8%, whereas only 3.6% of female participants reported concurrent use of the three aforementioned tobacco products (p<0.001). There was no difference in the prevalence of current hookah smoking only between males and females (2.2% vs 2.7%; p=0.141) (Table 3).

**Table 3:** Prevalence of single, dual, and triple current use (any use in past 30 days) of e-cigarettes, conventional cigarettes, and hookah in the total analytical sample and stratified by sex

*Tobacco product use*	*Total analytical sample (n=1525)*	*Males (n=694)*	*Females (n=831)*	p*
	*% (n)*	*% (n)*	*% (n)*	
None	64.9 (990)	41.6 (289)	84.4 (701)	<0.001
E-cigarettes only	4.0 (61)	5.9 (41)	2.4 (20)	<0.001
Cigarettes only	4.2 (64)	6.6 (46)	2.2 (18)	<0.001
Hookah only	2.4 (37)	2.2 (15)	2.7 (22)	0.141
E-cigarettes + Cigarettes	6.0 (92)	12.0 (83)	1.1 (9)	<0.001
E-cigarettes + Hookah	3.5 (54)	5.2 (36)	2.2 (18)	<0.001
Cigarettes + Hookah	2.1 (32)	2.7 (19)	1.6 (13)	0.001
E-cigarettes + Cigarettes + Hookah	12.8 (195)	23.8 (165)	3.6 (30)	<0.001
Any tobacco product use	35.1 (535)	58.4 (405)	15.6 (130)	<0.001
≥2 Tobacco products use	24.5 (373)	43.7 (303)	8.4 (70)	<0.001

* p-values comparing male and female proportions using chi-squared tests.

Prevalence estimates of exposure to household and public SHS/SHA are provided in Table 4. In total, exposure to household SHS and household SHA for ≥1 day in the past 7 days was reported by 41.9% and 32.0% of the total study participants, respectively. Daily exposure in the past 7 days to household SHS was higher than daily exposure to household SHA (23.3% vs 14.2%). Exposure to SHS/SHA in public places for ≥1 day in the past 7 days was reported by 62.2% of the study participants. In general, male participants were more often exposed to SHS/SHA compared to female participants (Table 4).

**Table 4:** Prevalence of exposure in the past 7 days to household secondhand smoke (SHS), household secondhand aerosols (SHA) from e-cigarettes, and public SHS and/or SHA in the total analytical sample and stratified by sex

*Exposure*	*Total analytical sample*	*Males*	*Females*	p*
	*% (n/total)*	*% (n/total)*	*% (n/total)*	
**Household SHS exposure**				
≥1 day	41.9 (619/1479)	48.5 (328/676)	36.2 (291/803)	<0.001
≥3 days	32.3 (477/1479)	37.4 (253/676)	27.9 (224/803)	<0.001
Every day	23.3 (344/1479)	26.0 (176/676)	20.9 (168/803)	0.020
**Household SHA exposure**				
≥1 day	32.0 (469/1465)	37.5 (251/669)	27.4 (218/796)	<0.001
≥3 days	22.5 (330/1465)	26.2 (175/669)	19.5 (155/796)	0.002
Every day	14.2 (208/1465)	15.3 (102/669)	13.3 (106/796)	0.292
**Public SHS/SHA exposure**				
≥1 day	62.2 (916/1472)	75.5 (507/672)	51.1 (409/800)	<0.001
≥3 days	44.8 (660/1472)	60.3 (405/672)	31.9 (255/800)	<0.001
Every day	26.8 (395/1472)	38.1 (256/672)	17.4 (139/800)	<0.001

* p-values comparing male and female proportions using chi-squared tests.

Associations between sex, age, and maternal and paternal educational level with current use of tobacco products and current exposure to SHS/ SHA were assessed (Table 5). Males were more likely than females to be current users of e-cigarettes (APR=5.19; 95% CI: 4.09–6.57), current smokers of conventional cigarettes (APR=5.42; 95% CI: 4.26– 6.90), and current smokers of hookah (APR=3.43; 95% CI: 2.72–4.32). Moreover, exposure to household SHS, household SHA, and public SHS/SHA was more common among males compared to females (Table 5). In general, age as well as maternal and paternal education were not associated with current tobacco product use and exposure to SHS/SHA (Table 5). However, study participants aged ≥18 years compared to those aged ≤16 years were more likely to be current cigarette smokers (APR=1.49; 95% CI: 1.19–1.87) (Table 5).

**Table 5:** Associations of demographic factors and parental education levels with adolescents’ current use (any use in past 30 days) of e-cigarettes, conventional cigarettes, and hookah as well as current exposure (any exposure in past 7 days) to household secondhand smoke (SHS), household secondhand aerosols (SHA), and public SHS/SHA

*Variables*	*Current use (any use in past 30 days)*			*Current exposure (any exposure in past 7days)*		
	*E-cigarettes*	*Cigarettes*	*Hookah*	*Household SHS*	*Household SHA*	*Public SHS/SHA*
	APR (95% CI)*	APR (95% CI)*	APR (95% CI)*	APR (95% CI)*	APR (95% CI)*	APR (95% CI)*
**Sex**						
Female	1.00 (Ref.)	1.00 (Ref.)	1.00 (Ref.)	1.00 (Ref.)	1.00 (Ref.)	1.00 (Ref.)
Male	5.19 (4.09–6.57)	5.42 (4.26–6.90)	3.43 (2.72–4.32)	1.33 (1.14–1.55)	1.30 (1.15–1.47)	1.46 (1.35–1.58)
**Age** (years)						
≤16	1.00 (Ref.)	1.00 (Ref.)	1.00 (Ref.)	1.00 (Ref.)	1.00 (Ref.)	1.00 (Ref.)
17	0.97 (0.80–1.19)	0.98 (0.79–1.22)	0.83 (0.65–1.06)	0.85 (0.71–1.02)	0.90 (0.78–1.04)	1.02 (0.93–1.13)
≥18	1.22 (0.98–1.54)	1.49 (1.19–1.87)	1.19 (0.91–1.55)	1.06 (0.86–1.31)	1.05 (0.88–1.24)	1.09 (0.97–1.22)
**Maternal education**						
Middle school or less	1.00 (Ref.)	1.00 (Ref.)	1.00 (Ref.)	1.00 (Ref.)	1.00 (Ref.)	1.00 (Ref.)
High school	1.19 (0.88–1.61)	1.178 (0.87–1.59)	1.14 (0.81–1.60)	0.88 (0.67–1.15)	0.90 (0.72–1.12)	1.01 (0.87–1.16)
Diploma^a^	1.24 (0.91–1.69)	1.33 (0.98–1.81)	0.95 (0.65–1.37)	0.90 (0.68–1.19)	0.84 (0.66–1.05)	1.05 (0.90–1.22)
Bachelor’s degree or higher	1.23 (0.91–1.66)	1.25 (0.93–1.68)	1.16 (0.83–1.63)	0.92 (0.71–1.19)	1.00 (0.81–1.24)	1.04 (0.90–1.19)
**Paternal education**						
Middle school or less	1.00 (Ref.)	1.00 (Ref.)	1.00 (Ref.)	1.00 (Ref.)	1.00 (Ref.)	1.00 (Ref.)
High school	0.83 (0.62–1.13)	0.88 (0.66–1.19)	0.77 (0.54–1.08)	1.02 (0.78–1.34)	0.90 (0.73–1.12)	0.99 (0.86–1.15)
Diploma^a^	0.79 (0.57–1.09)	0.86 (0.62–1.17)	0.93 (0.65–1.33)	0.85 (0.63–1.15)	0.92 (0.73–1.15)	0.98 (0.84–1.14)
Bachelor’s degree or higher	0.93 (0.70–1.24)	0.98 (0.74–1.31)	0.73 (0.52–1.02)	0.90 (0.69–1.18)	0.83 (0.67–1.03)	0.94 (0.82–1.09)

SHS: secondhand smoke. SHA: secondhand aerosol. APR: adjusted prevalence ratio. CI: confidence interval.

a Refers to a two-year associate degree post high school.

* Simultaneously adjusted for all variables shown in the table.

## DISCUSSION

This study estimated the prevalence and determined patterns of e-cigarette use, conventional cigarette smoking, and hookah smoking among adolescents in Kuwait for the first time. Moreover, the extent of household and public exposure to SHS and SHA from e-cigarettes among adolescents was assessed. In total, current (any use in the past 30 days) e-cigarette use, current conventional cigarette smoking, and current hookah smoking, was reported by 26.4%, 25.1%, and 20.9% of study participants, respectively. Overall, current use of any tobacco product and ≥2 tobacco products was reported by 35.3% and 24.3% of study participants, respectively. Frequency of concurrent triple current use of ‘e-cigarettes, cigarettes, and hookah’ was estimated to be 12.8% in the total study sample. Among the study participants, 41.9% reported current exposure to household SHS, 32.0% reported current exposure to household SHA from e-cigarettes, and 62.2% reported current exposure to SHS and/or SHA in public places. Male adolescents reported more usage of all of the assessed tobacco products and were more likely to report passive exposure to SHS/SHA than females. Results of this report add to the existing literature by showing, for the first time, that a large proportion of adolescents in Kuwait use e-cigarettes and hookah, in addition to conventional cigarettes. Also, the observation of elevated passive exposure to SHS/SHA among adolescents is new. Such findings can serve as baseline data to inform public health policies that aim to protect this vulnerable population.

In this report, the prevalence of current e-cigarette use was estimated to be 26.4%, which is higher than the 2018 estimate among high school students in the US of 20.8%^8^, but similar to the respective 2019 estimate of 27.5%^23^. Moreover, our estimate is also higher than estimates from Canada (14.6%) and England (8.9%) in 2018 among adolescents aged 16–19 years^9^. Comparing our estimated e-cigarette use prevalence to local estimates was not possible due to the lack of prior local estimates. In regard to conventional cigarette smoking, 25.1% of our study participants reported current use. This estimate is higher than estimates among adolescents in the US (8.1%), Canada (15.5%), and England (16.4%)^8,9^. However, the extent of conventional cigarette smoking in our study (25.1%) was similar to an estimated prevalence of 22.0% reported by the 2015 Global School-based Student Health Survey (GSHS) conducted among school students aged 13–17 years in Kuwait^25^, but higher than an estimated prevalence of 15.4% reported by the 2016 GYTS study conducted among school students aged 13–15 years in Kuwait^20^. Among our sample of high school students, prevalence of current hookah smoking was estimated to be 20.9%, which is much higher than what is reported among US high school students (4.1%)^8^, but lower than an estimated prevalence of 33.1% among male university students in Kuwait^26^. A global systematic review showed that hookah smoking is more common in the Eastern Mediterranean region in comparison to other regions, with estimates ranging from 2.5% in Oman to 37.2% in Lebanon^27^. Such increased use of hookah in this region of the world could be due to misperceptions of the related harms^28^.

Concurrent use of multiple tobacco products was ascertained in the current report. Among our study participants, 35.1% reported currently using at least one of the assessed tobacco products, while 24.5% reported concurrently using two or more of the three assessed products in the past 30 days. A study among US adolescents that assessed concurrent use of several tobacco products (i.e. e-cigarettes, cigarettes, cigars, smokeless tobacco, hookah, pipe tobacco, and/or bidis) estimated the prevalence of using two or more products to be 11.3%^8^, which is less than our estimate of 24.5%. The dual use of e-cigarettes and cigarettes was the most common (6.0%) dual smoking modality in the current report; this observation is similar to the results of a prior study among US adults, which reported that dual use of cigarettes and e-cigarettes is the most common dual smoking modality^29^. Triple use of e-cigarettes, cigarettes, and hookah was reported by 12.8% of our study participants, which is significantly higher than a respective estimate of 2.3% among

Serbian youth^30^. These findings indicate that a large proportion of high school students in Kuwait are concurrently using multiple tobacco products. The concurrent use of multiple tobacco products can be an indication of high nicotine dependence that needs to be addressed clinically and through social and psychological support. Moreover, such multiple use of tobacco products could increase the risk of tobaccorelated diseases among this young population. Hence, public health strategies, such as tobacco cessation programs, are needed to address this issue of multiple tobacco product use.

Our results indicate that a large proportion of the study participants are currently (past 7 days) exposed to household SHS, household SHA from e-cigarettes, and public SHS/SHA. Our estimate of household SHS exposure (41.9%) is higher than an estimate from the GYTS that found 30.4% of 356414 adolescents in 168 countries to be exposed to household SHS^31^, but lower than an estimate among adolescents living in low- and middle-income countries (55.9%)^5^. In the current study, 32.0% of the study participants reported exposure to household SHA from e-cigarettes in the past 7 days, which is similar to the 30-day SHA exposure among US high school students (29.0%)^17^. Exposure to SHS/SHA in public places in the past 7 days was reported by 62.2% of our study participants compared to 60.0% of US high school students^32^. The detrimental effects of exposure to SHS among neversmoking children and adults are well established^33^. In regard to SHA exposure, it has been shown that aerosol from e-cigarettes may contain potentially harmful substances, such as nicotine, heavy metals, ultrafine particulate matter, and volatile organic compounds^18^. Studies linking exposure to SHA from e-cigarettes with adverse health outcomes are emerging, with a recent study demonstrating an association between SHA exposure and asthma attacks among adolescents^34^. The findings of this report that a large proportion of adolescents in Kuwait are exposed to SHS/SHA are new and highlight a major threat to the health of adolescents.

In the current study, male participants reported significantly higher frequencies of current active smoking and passive exposure to smoking compared to female participants. For instance, males were more likely than females to be current e-cigarette users, current conventional cigarette smokers, and current hookah smokers. Additionally, exposure to household SHS, household SHA, and public SHS/ SHA were more common among males compared to females. Such sex related differences have been observed in prior studies^35,36^. Moreover, we did not observe associations between parental education level and adolescent smoking. This observation of no association is supported by findings of a prior report that indicated personal education among adolescents is related to their smoking behavior, and that parental education is not associated with adolescents smoking status^37^.

In addition to ratifying the WHO FCTC in 2006, Kuwait has enacted and enforced several laws that aim to prevent and reduce all forms of tobacco use in the population at large, for instance: increasing prices of tobacco products; banning use of tobacco products in all workplaces, public places, and on public transportation; prohibiting media promotions and advertising of all types of tobacco products; increasing the minimum age for purchasing tobacco products to 21 years; and requiring warning labels on all tobacco products^38^. Nevertheless, use of tobacco products is still substantial among adolescents in Kuwait as demonstrated by the results of this report. Several factors could contribute to such high use of tobacco products among adolescent, including the emergence of flavored tobacco in e-cigarettes and hookah, direct and indirect promotion and marketing through social media, misperceptions of harms, and wide access and availability^39,40^. In our study sample, of adolescents who are currently using tobacco products, 68.3% were not refused purchase of tobacco products because of their age during the past 30 days, and 51.4%, 39.5%, and 9.1% thought it is ‘easy’, ‘somewhat easy’, and ‘not easy at all’, respectively, for people at their age (i.e. minors) to purchase tobacco products in stores (data not shown). Such results indicate that tobacco products are widely accessible and available to minors in Kuwait. Hence, re-evaluations of the current national policies and enforcement of regulations and strategies are needed to reduce the burden of tobacco use among adolescents in Kuwait. Moreover, development and sustained implementation of evidence-based tobacco control strategies is essential to combat the ever diversifying tobacco products.

### Strengths and limitations

A major strength of the current study is the representative and large study sample, which allowed the estimation of tobacco product use prevalence among high school students throughout Kuwait. It is worth noting that school enrollment is very high in Kuwait for both females and males; hence, findings of our report can be extrapolated to the respective adolescent population in Kuwait. The high response rate (99.4%) is further evidence that self-selection bias was not an issue in our study. Another strength of the current study is that we have adapted questions from the standardized NYTS questionnaire, and followed similar definitions used by the NYTS standard^8^.

Nonetheless, our study had limitations. Tobacco product use was self-reported by participants, which is subject to recall and reporting bias. However, prior studies have shown that self-reported tobacco product use is closely related to objectively measured biomarkers of tobacco product use (e.g. cotinine levels)^41,42^. Hence, self-reporting of tobacco use can be considered as a valid measurement method that adds to the internal validity of our study. Another potential limitation is that we collected data from students enrolled in public high schools; therefore, generalization (external validity) of our findings might not be applicable to those enrolled in private schools or who have dropped out of school. A further limitation to our study is the lack of information on predictors of tobacco use.

## CONCLUSIONS

The current study demonstrated that a large proportion of adolescents in Kuwait are current users of e-cigarettes, conventional cigarettes, and/or hookah. Similarly, exposure to SHS and/or SHA in households and public places is very common. E-cigarette use was the most commonly reported smoking modality followed by conventional cigarettes among our study participants. These findings indicate that patterns of tobacco product use among adolescents in Kuwait follow international trends, where e-cigarettes are gaining substantial popularity among youth. Moreover, we observed that a considerable proportion of our study participants concurrently use multiple tobacco products. Collectively, high proportions of active smoking and passive exposure to smoking is observed among high school students in Kuwait. Such an alarming observation calls for public health policies and strategies that aim to reduce smoking in the community at large and denormalize all types of smoking. Also, continued surveillance of all forms of tobacco product use among youth and identification of associated factors are important elements of successful control and prevention strategies.
